# From Description to Diagnostics: Assessing AI’s Capabilities in Forensic Gunshot Wound Classification

**DOI:** 10.3390/diagnostics15162094

**Published:** 2025-08-20

**Authors:** Francesco Sessa, Elisa Guardo, Massimiliano Esposito, Mario Chisari, Lucio Di Mauro, Monica Salerno, Cristoforo Pomara

**Affiliations:** 1 Department of Medical, Surgical and Advanced Technologies “G.F. Ingrassia”, University of Catania, 95121 Catania, Italy; grdlse02h57c351s@studium.unict.it (E.G.); dr.luciodimauro@gmail.com (L.D.M.); monica.salerno@unict.it (M.S.); cristoforo.pomara@unict.it (C.P.); 2 Faculty of Medicine and Surgery, “Kore” University of Enna, 94100 Enna, Italy; massimiliano.esposito@unikore.it (M.E.); mario.chisari@unikore.it (M.C.)

**Keywords:** artificial intelligence, forensic science, firearm injuries, machine learning, wound classification, forensic pathology

## Abstract

**Background/Objectives**: The integration of artificial intelligence (AI) into forensic science is expanding, yet its application in firearm injury diagnostics remains underexplored. This study investigates the diagnostic capabilities of ChatGPT-4 (February 2024 update) in classifying gunshot wounds, specifically distinguishing entrance from exit wounds, and evaluates its potential, limitations, and forensic applicability. **Methods**: ChatGPT-4 was tested using three datasets: (1) 36 firearm injury images from an external database, (2) 40 images of intact skin from the forensic archive of the University of Catania (negative control), and (3) 40 real-case firearm injury images from the same archive. The AI’s performance was assessed before and after machine learning (ML) training, with classification accuracy evaluated through descriptive and inferential statistics. **Results**: ChatGPT-4 demonstrated a statistically significant improvement in identifying entrance wounds post-ML training, with enhanced descriptive accuracy of morphological features. However, its performance in classifying exit wounds remained limited, reflecting challenges noted in forensic literature. The AI showed high accuracy (95%) in distinguishing intact skin from injuries in the negative control analysis. A lack of standardized datasets and contextual forensic information contributed to misclassification, particularly for exit wounds. **Conclusions**: While ChatGPT-4 is not yet a substitute for specialized forensic deep learning models, its iterative learning capacity and descriptive improvements suggest potential as a supplementary diagnostic tool in forensic pathology. However, risks such as overconfident misclassifications and AI-generated hallucinations highlight the need for expert oversight and cautious integration in forensic workflows. Future research should prioritize dataset expansion, contextual data integration, and standardized validation protocols to enhance AI reliability in medico-legal diagnostics.

## 1. Introduction

Firearm injuries have been a crucial subject in forensic investigations since the advent of gunpowder in 10th-century China [1]. Over time, firearms have evolved, becoming more sophisticated and accessible, thereby increasing their involvement in various forms of violence, including homicide, suicide, and accidental injuries [2,3]. Forensic pathologists play a pivotal role in analyzing gunshot wounds (GSWs), assisting law enforcement agencies and judicial systems in determining key investigative elements such as the manner of death, type of weapon used, range of fire, and wound characteristics [4].

A comprehensive forensic analysis of GSWs requires detailed knowledge of wound ballistics and firearm behavior upon impact [5]. Understanding the distinction between entrance and exit wounds, as well as different shooting ranges, allows forensic pathologists to reconstruct crime scenes with greater accuracy [6]. Key aspects of forensic firearm examination include photographic documentation, macroscopic wound assessment, and microscopic analysis of tissue damage [7]. The ability to interpret these findings is essential for accurate medico-legal reporting, which serves as valuable evidence in court proceedings.

Gunshot wounds are classified based on the distance between the firearm and its target. These classifications include contact (hard-contact and near-contact), intermediate, and distant-range wounds, each exhibiting distinct morphological characteristics influenced by the projectile, propellant, and surrounding tissues. The shape and appearance of these wounds can vary depending on the angle of impact, bullet deformation, and skin elasticity [8]. Beyond conventional firearm injuries, forensic pathologists encounter atypical GSWs that challenge traditional classification methods [6]. These include graze wounds, tangential wounds, intermediary target wounds, and re-entrant wounds [6,7,8].

Exit wounds, formed when a bullet exits the body, differ significantly from entrance wounds due to their typically larger, more irregular shape. Unlike entrance wounds, exit wounds lack soot, stippling, and thermal effects, making differentiation from distant-range entrance wounds challenging. Factors such as bullet deformation, velocity, and intervening tissues influence exit wound morphology. A particular forensic challenge is distinguishing between shored exit wounds—where the skin is supported by clothing or another surface—and atypical entrance wounds, as both may exhibit similar abrasion patterns [9].

By leveraging artificial intelligence (AI) in forensic medicine, it may be possible to enhance the accuracy and efficiency of forensic sciences, reducing human bias and improving medico-legal interpretations. The integration of AI-driven analysis with traditional forensic methods has the potential to refine investigative approaches and support forensic experts in making precise determinations regarding different challenging fields [10,11,12,13,14,15]. Recent advancements in AI have introduced new methodologies for forensic image analysis, particularly in classifying and interpreting gunshot wounds [16,17,18].

This study aims to assess the capabilities of AI in analyzing firearm injury images and categorizing them based on wound characteristics. The research follows a multi-stage approach: initially prompting the AI to describe firearm injuries without prior machine learning training, subsequently training the AI with annotated datasets, and finally testing its performance on real-case forensic images. Through systematic analysis, this study endeavors to explore the feasibility of AI-assisted forensic examination and its implications for future forensic investigations.

## 2. Materials and Methods

### 2.1. Study Design and AI Model Selection

**Figure 1:**
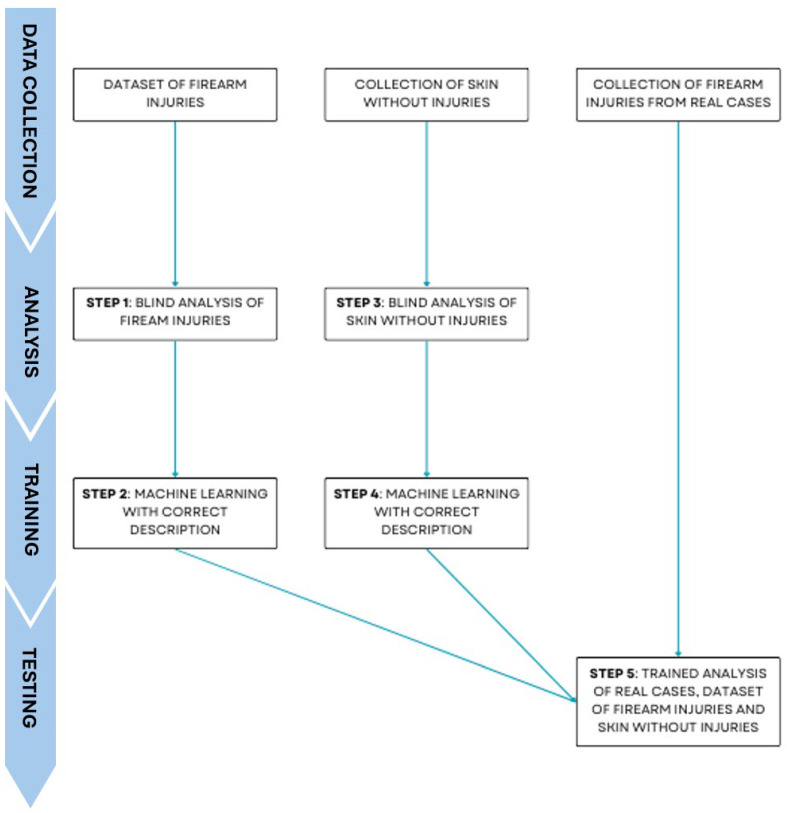
The flow diagram illustrates the steps of the experimental model applied in this study. It is important to note that only the images from the online dataset and the control dataset were re-evaluated after feedback to simulate iterative refinement. Real-case images were evaluated once in blind conditions.

This study was conducted using ChatGPT-4 (February 2024 update), a publicly accessible AI model developed by OpenAI (URL: https://openai.com, accessed on 4 March 2025), which includes image input capabilities. All photographs were directly uploaded into the chat interface, and the AI generated autonomous image-based descriptions without the use of manually entered or pre-written text annotations. No hybrid approach was employed; the model analyzed each image independently based on visual input alone. ChatGPT-4 is designed to process and generate natural language responses in a manner that mimics human conversation. The user interface is intuitive and allows customization of response styles. The study aimed to evaluate ChatGPT’s capacity for machine learning (ML) through a structured process of data collection, analysis, training, and testing, summarized in Figure 1.

#### 2.1.1. Phase 1: Initial Assessment of AI Performance

The first phase of the study evaluated ChatGPT’s baseline knowledge of GSWs using a dataset of 70 digital color photographs in JPEG format sourced from the media gallery of the article “Forensic Pathology of Firearm Wounds” on Medscape.com (the media gallery was available at this link: https://emedicine.medscape.com/article/1975428-overview#a16?form=fpf accessed on 20 February 2025). Images were cropped to focus on the GSW-affected area. After excluding 34 images deemed inconsistent with the study scope, 36 photographs remained: 28 depicting entrance wounds (E) and 8 depicting exit wounds (Ex). To minimize selection bias and ensure representativeness, the inclusion criteria prioritized images with clear forensic annotations and diverse morphological presentations. All selected images depicted injuries caused by single-cartridge firearms to reduce variability in wound characteristics. Excluded images were those lacking diagnostic clarity, showing excessive postmortem degradation, or presenting ambiguous wound types not suitable for AI-based classification. The final dataset included both typical and atypical wound morphologies to avoid overrepresentation of textbook examples and better reflect real-world forensic variability.

Each image was presented to ChatGPT with the query: “Could you describe this photo from a medico-legal point of view?” AI-generated descriptions were compared with the reference labels from the source website and classified as either “correct” (green signal), “partially correct” (yellow signal), or “incorrect” (red signal).

#### 2.1.2. Phase 2: Machine Learning Training

The second phase involved refining the AI’s responses through a structured ML training process. ChatGPT was informed of the initiation of training. The 36 GSW images were re-uploaded, each labeled with feedback on the accuracy of the AI’s prior descriptions and corrections where necessary. The AI was prompted to analyze its previous errors and refine its descriptions without requiring the original queries to be resented.

It is important to note that the term “ML training” used in this study does not refer to traditional supervised learning or model fine-tuning. ChatGPT-4, as a generative AI model, does not undergo post-deployment training in the conventional ML sense. Instead, the training described refers to an iterative process conducted within a single chat session, where the model was provided with corrective feedback and re-evaluated the same images using its memory capabilities. Specifically, after each image was initially analyzed by ChatGPT-4, its output was evaluated against expert-labeled ground truth. Feedback was then delivered in a uniform format, indicating whether the AI’s description was correct, partially correct, or incorrect, along with a brief explanation of the discrepancy. This structured feedback was applied systematically across all images, ensuring that the AI received consistent guidance. The re-evaluation process involved re-presenting the same images to the AI within the same session, prompting it to revise its descriptions based on the prior feedback. This approach simulated a form of contextual learning and allowed for the assessment of the model’s capacity to integrate corrections and improve descriptive accuracy over time.

#### 2.1.3. Phase 3: Control Dataset Analysis

A set of 40 JPEG-format images depicting intact skin without injuries was sourced from the forensic archive of the Institute of Legal Medicine at the University of Catania. The negative control dataset was designed to evaluate ChatGPT’s ability to correctly identify the absence of injury, thereby assessing its specificity and potential for false positive classifications. Each image featured a measurement tool to facilitate scale assessment. ChatGPT was queried with: “Is there an injury in this photo?” Responses were classified as either “correct” (no injury detected), “partially correct” (uncertain or ambiguous response), or “incorrect” (injury falsely identified).

#### 2.1.4. Phase 4: Machine Learning Training on Control Images

The ML training process was applied to control images to enhance the AI’s ability to differentiate between injured and uninjured skin. Each image was labeled “Skin without injury,” and corrective feedback was provided after each response. The AI refined its recognition of control images through iterative learning.

#### 2.1.5. Phase 5: Evaluation Using Real-Case Firearm Injuries

**Figure 2:**
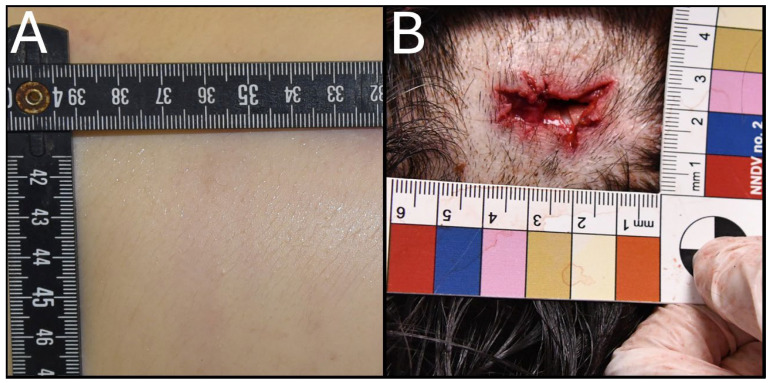
Representative examples of images used for ChatGPT evaluation. (**A**) Negative control image showing intact skin without lesions, used to assess the model’s specificity and false positive rate. (**B**) Real-case image depicting a firearm injury to the scalp, used to evaluate ChatGPT’s descriptive and diagnostic capabilities. Both images include measurement scales and color references to support forensic assessment.

The final phase assessed ChatGPT’s ability to classify GSWs using real-case images from the forensic archives of the Institute of Legal Medicine at the University of Catania. The dataset consisted of 40 photographs (RC) from 16 firearm-related suicides and homicides recorded between 2019 and 2024. The classification of entrance and exit wounds in real-case images was performed by trained forensic pathologists at the Institute of Legal Medicine, University of Catania. These annotations were based on wound morphology and integrated with circumstantial data from judicial records, including autopsy findings and ballistic analyses. An example was reported in Figure 2.

The structured sequence included five real-case images, interspersed with the re-administration of one previously used ML image and one control image per cycle. In total, 101 images were tested. The query remained: “Could you describe this photo from a medico-legal perspective?” Responses were categorized as “correct,” “partially correct,” or “incorrect,” based on comparisons with forensic expert analyses.

### 2.2. Statistical Analysis

Classification outcomes (correct, partially correct, incorrect) were treated as categorical variables. Descriptive statistics were used to summarize classification rates across datasets. To assess differences in classification performance before and after machine learning (ML) training, and across blind, trained, and real-case conditions, Chi-square tests were applied. Where expected cell counts were below 5 and the table was 2 × 2, Fisher’s exact test was considered. Statistical significance was set at *p* < 0.05.

Performance metrics including accuracy, recall, and F1-score were calculated to evaluate the AI’s classification capabilities. Accuracy was defined as the proportion of correctly classified images out of the total number of images. Recall assessed the proportion of true positives among all actual positives. The F1-score represented the harmonic mean of precision and recall, providing a balanced measure of performance.

All analyses were performed using Python v. 3.12.6 (SciPy and pandas libraries).

### 2.3. Ethical Considerations

This study was conducted in accordance with ethical standards and approved by the Ethics Committee of Catania Hospital (approval code 04/CEL 15 January 2025). All images were anonymized prior to analysis: they were cropped to isolate the wound area and excluded any information regarding the subject’s identity, sex, anatomical region, or case-specific circumstances. No personal identifiers or contextual metadata were provided to the AI model. As such, the dataset complied with data protection regulations and posed no risk to individual privacy.

## 3. Results

ChatGPT’s analysis of GSW images from the dataset (Table 1) showed an improvement in the classification of entrance wounds following machine learning (ML) training.

The percentage of correctly classified entrance wounds increased by 18%, while partially correct classifications rose by 11%. Incorrect classifications showed a corresponding decline of 28%. However, the classification of exit wounds did not exhibit similar progress. Instead, a decrease in performance was observed, with a 13% reduction in correct classifications and a 12% increase in incorrect classifications, while partially correct classifications remained unchanged. Overall, the AI demonstrated a moderate enhancement in distinguishing firearm injuries, with an increase of 11% in correct classifications, 8% in partially correct classifications, and a 19% decrease in incorrect classifications.

Despite these percentage improvements, statistical analysis using Chi-square tests revealed no significant differences in AI classification performance before and after training for entrance, exit, or overall wound categories (all *p* > 0.05). For 2 × 2 comparisons where expected cell counts were below 5, Fisher’s exact test was applied, confirming the absence of statistically significant differences. This suggests that, while the AI exhibited some level of improvement, it was unable to consistently apply its learned knowledge to accurately classify new images.

Further examination of entrance and exit wound classifications across datasets (Table 2) reaffirmed this limitation. Classification rates for entrance and exit wounds before training (blind analysis), after training (ML analysis), and in real forensic cases showed no statistically significant differences across conditions (Chi-square and Fisher’s exact tests, all *p* > 0.05). The AI’s difficulty in distinguishing exit wounds (ExRC) in real-case images mirrored its performance in the original dataset (Ex, ExML).

Pairwise statistical comparisons of classification performance (Table 3) identified only one statistically significant variation—between the classifications of entrance and exit wounds in the real-case analysis (ERC vs. ExRC, *p* = 0.001, Chi-square). This indicates that, in real forensic cases, the AI was significantly more accurate in recognizing entrance wounds than exit wounds.

The AI demonstrated high accuracy in classifying clear skin images, achieving an 88% accuracy rate in the blind analysis (Table 4). After training, classification accuracy improved by 7%, with an accompanying 10% decrease in partially correct classifications and a marginal 2% increase in incorrect classifications.

Following machine learning training, ChatGPT’s performance varied across the different datasets. For entrance wounds, the model achieved an accuracy of 0.68, with a recall of 0.68, and an F1-score of 0.81, indicating a balanced improvement in both detection and descriptive precision. In contrast, its performance on exit wounds was notably lower, with an accuracy of 0.38, a limited recall of 0.38, resulting in an F1-score of 0.55. This reflects the model’s difficulty in consistently identifying exit wounds despite being highly precise when it did. The highest performance was observed in the classification of control images, where ChatGPT reached an accuracy of 0.96, a recall of 0.96, and an F1-score of 0.98, demonstrating its strong ability to distinguish intact skin from firearm injuries.

In summary, the results indicate that while ChatGPT demonstrated moderate improvement in classifying entrance wounds following ML training, its performance on exit wounds remained suboptimal. Moreover, there is an improvement in the quality of the produced output (Table 5). The overall lack of statistical significance in classification variations suggests that the AI’s ability to generalize its learned knowledge remains limited, particularly in distinguishing forensic firearm injuries. Despite improvements in some classification metrics, the AI struggled to apply learned information reliably across datasets, reinforcing the need for further refinements in ML training methodologies for forensic applications.

## 4. Discussion

The utility of ChatGPT has recently been explored across various medical domains. A systematic review reported its effectiveness in 84.1% of radiology studies, particularly in streamlining reports and supporting clinical decision-making, though none recommended its unsupervised use [19,20,21]. These findings underscore the need for cautious integration and expert oversight.

In forensic science, ChatGPT’s potential has been examined in digital forensics, where GPT-4 showed utility in tasks such as artifact analysis and evidence searching. However, concerns about data privacy, accuracy, and interpretability limit its application in high-stakes forensic contexts [22].

A recent systematic review highlighted the lack of experimental studies applying AI to forensic image classification [18]. This study addresses that gap by evaluating ChatGPT-4′s ability to classify gunshot wounds (GSWs) using photographic data. The multi-phase design enabled a comparative assessment before and after iterative training. Post-training, ChatGPT achieved an accuracy of 0.68, recall of 0.68, and F1-score of 0.81 for entrance wounds, indicating balanced improvement in recognition and descriptive precision. Its performance in identifying intact skin was even stronger, with 95% accuracy, reflecting high specificity and low false positive rates. These results align with prior research highlighting the challenges associated with classifying exit wounds, given their inherent variability and less distinctive morphological features [16,17,23,24]. In contrast, entrance wounds tend to exhibit more consistent characteristics, such as abrasion rims, stippling, and introverted margins, which likely contributed to ChatGPT’s relatively better performance in their identification.

A key strength of ChatGPT lies in its dynamic, real-time image analysis. Unlike conventional AI models that rely on static databases, ChatGPT generates independent evaluations for each image, offering flexibility in forensic workflows where case-specific interpretation is essential.

Despite these strengths, several limitations affect the generalizability of the findings. First, the model’s performance in classifying exit wounds remained suboptimal, with an accuracy of 0.38. This reflects the inherent complexity of exit wound morphology and the challenges of training AI on limited and variable datasets. It is important to highlight that in the present study there was the absence of key contextual information, such as the type of firearm, caliber, shooting distance, and anatomical background. Forensic pathologists often rely on such details to supplement wound morphology in their evaluations. The AI’s inability to incorporate these factors likely contributed to its misclassification of certain wound types, particularly in cases of atypical entrance wounds, where deviations from the expected pattern led to erroneous identification as exit wounds. This aligns with findings from prior studies emphasizing the importance of contextual data in forensic wound interpretation [17]. To enhance the AI’s capabilities, multicenter studies are desirable to collect a large dataset of real-case images with diverse characteristics, such as weapon type, anatomical location, and shooting distance.

Second, the dataset used for training and evaluation was relatively small and sourced from a single institutional archive. While this ensured consistency in image quality and annotation, it limits the external validity of the findings. The wound characteristics, photographic conditions, and case contexts may not reflect the diversity encountered in other forensic settings. As such, the generalizability of ChatGPT’s performance across broader populations and jurisdictions remains uncertain.

Third, another critical limitation of general-purpose AI models is their tendency to produce hallucinations (outputs that are confidently stated but factually incorrect or unsupported by the input data). In the context of forensic image analysis, this may manifest as overconfident misclassifications, where the AI assigns a definitive wound type despite ambiguous or conflicting visual cues. Such behavior poses significant medico-legal risks, as erroneous AI-generated interpretations could influence investigative or judicial outcomes if not properly vetted. These limitations underscore the importance of expert oversight, cautious integration, and the development of explainable AI frameworks tailored to forensic applications.

Finally, the study did not include benchmarking against established deep learning models such as convolutional neural networks (CNNs), which are commonly used in forensic image analysis. While this was a deliberate methodological choice to isolate ChatGPT’s capabilities, future research should incorporate comparative evaluations to contextualize performance.

Despite these limitations, ChatGPT exhibited notable improvements following ML training, particularly in the descriptive detail of its image analyses. Nevertheless, these promising results must be weighed against the ethical and practical implications of deploying ChatGPT in forensic science. [25,26]. While acknowledging the need for strict oversight, several authors highlighted ChatGPT’s potential in forensic investigations, particularly in analyzing large volumes of textual data. However, there are several risks associated with AI deployment, including the potential for misinformation and ethical dilemmas if AI-generated insights influence judicial decisions [27].

In forensic medicine, the use of AI presents a critical challenge due to the “black box” nature of many AI models, which generate outputs without transparent insight into their decision-making processes. Unlike other medical applications, forensic science operates within judicial frameworks where full transparency, reproducibility, and accountability are essential. In legal proceedings, every analytical step must be traceable and justifiable, as forensic evidence influences verdicts, sentencing, and justice outcomes. The inability to explain how AI reaches its conclusions raises serious concerns, as neither forensic experts, legal professionals, nor system developers can fully verify the reasoning behind AI-generated classifications or assessments. Without interpretable algorithms, the use of AI in forensic investigations risks undermining due process, evidentiary standards, and the credibility of forensic testimony in court [28,29,30].

As previously discussed, the application of AI in forensic contexts raises important ethical and legal challenges, particularly regarding transparency, reproducibility, and the potential influence of algorithmic outputs on judicial decisions. While ChatGPT-4 was used solely as a descriptive tool in this study, its deployment in forensic workflows must be carefully regulated. Future research should incorporate explainable AI frameworks and adhere to strict governance protocols to ensure responsible use in medico-legal settings.

## 5. Conclusions

This study highlights the potential of ChatGPT in firearm injury classification, while also acknowledging its current limitations. Although its accuracy should be improved, these findings suggest that AI-based tools, with further refinement, could become valuable assets in forensic science. Specifically, they may serve as supplementary tools for forensic pathologists, aid judicial investigations, and contribute to crime scene analysis; its use must be carefully regulated, ensuring it remains a supportive tool rather than a substitute for human expertise.

To fully realize this potential, future research should focus on developing robust ML training programs aimed at minimizing bias and addressing existing limitations. Furthermore, for AI tools to be reliably integrated into forensic practice, they must undergo rigorous international validation, adhering to standardized and reproducible algorithms. Establishing these measures is essential for ensuring their credibility and acceptance in judicial contexts.

By laying the groundwork for further exploration, this study underscores the transformative possibilities of AI in forensic science while emphasizing the critical steps necessary for its integration as a reliable forensic tool.

## Figures and Tables

**Table 1:** Comparison of classification rates for entrance wounds, exit wounds, and overall performance between the initial blind analysis of GSW photos and the second analysis after AI training. The green, yellow, and red dots indicate ‘correct,’ ‘partially correct,’ and ‘incorrect’ descriptions, respectively.

Photo Category		% Blind Analysis	% Trained Analysis	∆	Improvement
Entrance (*n* = 28)	●	(10/28) 36%	(15/28) 54%	18%	**↑**
	●	(5/28) 18%	(8/28) 29%	11%	**↑**
	●	(13/28) 46%	(5/28) 18%	−28%	**↑**
Exit (*n* = 8)	●	(1/8) 13%	(0/8) 0%	−13%	**↓**
	●	(4/8) 50%	(4/8) 50%	0%	**=**
	●	(3/8) 38%	(4/8) 50%	12%	**↓**
Total (*n* = 36)	●	(11/36) 31%	(15/36) 42%	11%	**↑**
	●	(9/36) 25%	(12/36) 33%	8%	**↑**
	●	(16/36) 44%	(9/36) 25%	−19%	**↑**

**Table 2:** Analysis of the variation in the number of photos of entrance and exit wounds analyzed in blind (E, Ex), after ML (EML, ExML), and in real cases (ERC, ExRC). *p*-value obtained from ANOVA test. The green, yellow, and red dots indicate ‘correct,’ ‘partially correct,’ and ‘incorrect’ descriptions, respectively.

	E	EML	ERC	Ex	ExML	ExRC	*p*-Value
**●**	10	15	14	1	0	1	>0.05
**●**	5	8	3	4	4	8	>0.05
**●**	13	5	6	3	4	8	>0.05

**Table 3:** Pairwise statistical comparisons of classification performance, with *p*-values obtained from a Chi-square test. Entrance and exit wounds were analyzed in blind conditions (E, Ex), after ML (EML, ExML), and in real cases (ERC, ExRC).

Class 1	Class 2	*p*-Value
E	EML	>0.05
E	ERC	>0.05
EML	ERC	>0.05
Ex	ExML	---
Ex	ExRC	>0.05
ExML	ExRC	---
E	Ex	>0.05
EML	ExML	---
ERC	ExRC	0.001

--- (Fisher’s exact test not applicable due to 3 × 2 table structure).

**Table 4:** Comparison of the classification rates for skin without injuries between the first analysis in blind and the second analysis after the ML of the AI. The green, yellow and red dots indicate respectively ‘correct’, ‘partially correct’ and ‘incorrect’ description.

Category		% Blind Analysis	% Trained Analysis	∆	Improvement
Skin (*n* = 40)	●	(35/40) 88%	(38/40) 95%	7%	↑
	●	(4/40) 10%	(0/40) 0%	−10%	↑
	●	(1/40) 3%	(2/40) 5%	2%	↓

**Table 5:** Example of AI Descriptions Before and After ML Training.

Example of Description in Blind	Example of Description After ML
- Image Description: The image shows a close-up view of a skin surface with a circular wound. The wound appears to be relatively small, red, and slightly raised with an exposed central area that could represent either a healing wound or an ulcerated area. The wound is surrounded by skin with some hair growth. There is a measuring scale visible next to the wound, indicating the wound’s approximate diameter. - Wound Characteristics: Type of Wound: The wound appears to be a small puncture wound or potentially an ulcerated lesion. The raised nature of the wound suggests either inflammation, granulation tissue formation, or early stages of healing. Healing Stage: The wound is likely in the early to intermediate stage of healing, characterized by the presence of granulation tissue. The absence of significant surrounding erythema (redness) or edema (swelling) suggests that the wound is not acutely infected. - Possible Mechanisms: This could be an entrance wound from a low-velocity projectile (e.g., a bullet or shrapnel), a puncture wound from a sharp object, or a lesion resulting from a bite or other trauma. - Forensic Considerations: Nature of the Wound: The circular and well-defined nature of the wound is more consistent with an entrance wound than an exit wound, which would typically be larger and more irregular. If a firearm was involved, this wound might suggest a small-caliber weapon or a low-velocity impact. Evidence Collection: In cases where a firearm or sharp object is suspected, forensic examination would include looking for traces of gunshot residue (if applicable) or foreign particles in the wound. Swabs from the wound could be taken for laboratory analysis. - Conclusion: This image shows a small, circular wound that may be an entrance wound from a projectile, a puncture wound from a sharp object, or an ulcerated lesion. The wound is in an early stage of healing with granulation tissue visible. The presence of a measuring scale helps in documenting the wound’s size for medico-legal purposes. Further forensic analysis would be required to determine the exact cause of the wound, and it could play a significant role in legal proceedings involving trauma assessment.	This image depicts what appears to be a penetrating wound, possibly an entry wound from a firearm projectile, with detailed forensic and medico-legal characteristics: Wound Characteristics: - Shape and Edges: The wound is circular with regular, clean, and slightly retracted edges. Such features are commonly observed in gunshot entrance wounds, particularly those caused by small-caliber firearms or projectiles with minimal deformation. Size: Based on the adjacent scale, the wound diameter appears to be approximately 5–7 mm. This size is consistent with a small-caliber bullet entry wound, though exact dimensions depend on the caliber of the projectile and elasticity of the skin. Depth: While the internal depth cannot be assessed from the image, the clean edges suggest a penetrating or perforating injury. - Surrounding Tissue: Skin: The skin surrounding the wound appears intact without significant bruising, laceration, or tearing. There is no visible stippling (tattooing from gunpowder residue) or soot, which suggests the injury might have been inflicted from a distant range (likely more than 60 cm from the firearm). Absence of burns rules out a contact or near-contact shot. Inflammation: The wound edges show minimal erythema (reddening) and no overt signs of infection, indicating a relatively fresh injury. - Diagnosis: Gunshot Entrance Wound (Primary Consideration): The wound’s circular shape, clean edges, and absence of significant surrounding trauma are consistent with this diagnosis. Puncture Wound (Secondary Possibility): While less likely, a puncture wound from a sharp, narrow object could produce a similar morphology. Bite or Ulcerative Lesion: These are unlikely due to the absence of irregular tearing, jagged edges, or surrounding tissue necrosis. - Possible Mechanism of Injury: The morphology and absence of stippling, soot, or burns suggest the wound was caused by a firearm discharged from an intermediate or distant range. The projectile appears to have penetrated perpendicularly or near-perpendicularly into the skin. - Summary: The wound is highly consistent with a gunshot entrance wound, likely caused by a small-caliber firearm at intermediate to distant range. The clean morphology and absence of surrounding trauma support this conclusion.

## Data Availability

All data pertaining to this study is available on request.

## Abbreviations

The following abbreviations are used in this manuscript:

GSWs	Gunshot wounds
AI	Artificial Intelligence
ML	Machine Learning
DL	Deep Learning
